# The Role of Immunonutrients in the Prevention of Necrotizing Enterocolitis in Preterm Very Low Birth Weight Infants

**DOI:** 10.3390/nu7095334

**Published:** 2015-08-28

**Authors:** Ping Zhou, Yanqi Li, Li-Ya Ma, Hung-Chih Lin

**Affiliations:** 1 Department of Neonatology, Bao’an Maternal and Child Health Hospital, Shenzhen 518133, Guangdong, China; E-Mails: xianggalao@126.com (P.Z.); maria226@sina.com (L.-Y.M.); 2 Comparative Pediatrics and Nutrition, University of Copenhagen, Frederiksberg DK-1870, Denmark; E-Mail: yli@sund.ku.dk; 3 Children’s Hospital of China Medical University, No. 2 Yuh Der Road, Taichung 404, Taiwan; 4 School of Chinese Medicine, China Medical University, No. 91 Hsueh-Shih Road, Taichung 404, Taiwan

**Keywords:** necrotizing enterocolitis, very low birth weight infants, prevention, nutrients, probiotics

## Abstract

Necrotizing enterocolitis (NEC) is a critical intestinal emergency condition, which mainly occurs in preterm very low birth weight (PVLBW) infants. Despite remarkable advances in the care of PVLBW infants, with considerable improvement of the survival rate in recent decades, the incidence of NEC and NEC-related mortality have not declined accordingly. The fast progression from nonspecific signs to extensive necrosis also makes primary prevention the first priority. Recently, increasing evidence has indicated the important role of several nutrients in primary prevention of NEC. Therefore, the aim of this review is to summarize some potential immunomodulatory nutrients in the prevention of NEC, including bovine colostrum, probiotics, prebiotics (e.g., human milk oligosaccharides), long chain polyunsaturated fatty acids, and amino acids (glutamine, cysteine and *N*-acetylcysteine, l-arginine and l-citrulline). Based on current research evidence, probiotics are the most documented effective method to prevent NEC, while others still require further investigation in animal studies and clinical randomized controlled trials.

## 1. Introduction

**Figure 1:**
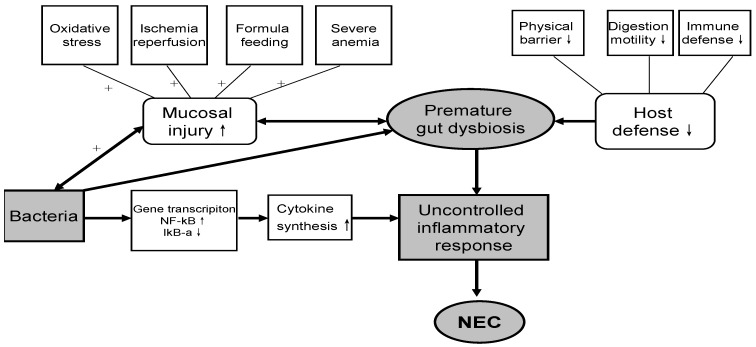
Possible mechanisms involved in the pathogenesis of NEC.

Necrotizing enterocolitis (NEC) is an acute and potentially fatal disease characterized by inflammation and necrosis in the gastrointestinal tract (GIT). Incidence of NEC is between 7% and 12% in preterm very low birth weight (PVLBW) infants with an estimated mortality of 15%–30% [1]. The pathogenesis of NEC is still incompletely understood, but it is thought that several factors are involved interactively, such as premature birth, low birth weight, ischemia/reperfusion (I/R) injury, abnormal gut bacterial colonization, and inappropriate enteral feeding [2] (Figure 1). Due to the obscure multifactorial etiology, early diagnosis and effective treatment of NEC is limited. Consequently, effective strategies in the prevention of NEC are critically needed.

Many intestinal functions are affected by prematurity, which may predispose preterm infants to NEC. These include GIT motility, mesenteric blood flow, digestive and absorptive functions, mucosal barrier function, bacterial colonization, and gut immunity. Feeding strategies and nutritional interventions play important roles in the modulation of these functions and thus the prevention of NEC [3]. Human milk feeding has been shown to prevent NEC [4], whereas infant formula (IF) feeding is associated with a higher risk of NEC, with a typical risk ratio of 2.77 (95% confidence interval (CI) 1.40 to 5.46) [5]. The superiority of human milk may be attributed to nutrients that can modulate the intestinal digestive function, barrier function, bacterial colonization, and host immune defense, known as immunonutrients [6]. Understanding of these nutrients is important in developing better nutritional support for preterm infants, especially for those with limited access to human milk. Even in developed countries like UK, Ireland, Canada, Australia, and New Zealand, only 7%–65% neonatal intensive care units have access to human donor milk [7]. To summarize immunonutrients that may have clinical potential in the prevention of NEC, a literature search was performed in PubMed, Embase, and Chinese Biomedical Literature (CBM) databases with the focus on the following nutrients: bovine colostrum, probiotics, human milk oligosaccharides, long chain polyunsaturated fatty acids, and amino acids (*i.e.*, glutamine, cysteine and *N*-acetylcysteine, l-arginine and l-citrulline). Both animal studies and clinical trials were reviewed to provide consideration of the clinical potentials of each nutrient. The suggested NEC-preventive mechanisms of these nutrients are summarized in Table 1.

**Table 1:** NEC-preventive mechanisms.

Nutrients	Anti-Inflammation	Anti-Oxidant Stress	Regulating Intestine Blood Flow	Immuno-Regulation	Improving Gut Bacterial Colonization	Improving Intestine Cell Growth and Development
Bovine colostrum	+	−	−	+	+	+
Probiotics	−	−	−	+	+	+
HMOs and Prebiotics	+	−	−	+	+	−
LCPUFAs	+	+	−	+	−	−
Glutamine	+	+	−	−	−	+
Cysteine, NAC	+	+	−	−	−	−
Arginine/citrulline	+	+	+	−	−	−

HMOs, Human milk oligosaccharides; LCPUFAs, *n-*3 long chain polyunsaturated fatty acids; NAC, *N*-acetylcysteine.

## 2. Bovine Colostrum

Bovine colostrum (BC) is the early milk from cows, which contains much higher amounts of trophic and immuno-regulatory factors than those in IF (e.g., insulin-like growth factor, epidermal growth factor, immunoglobulins, lactoferrin, transforming growth factor-β). These factors exert various physiological functions including intestinal growth and development, intestinal defense, immuno-regulation, and anti-infection [8]. The effect of BC in the prevention of NEC has been repeatedly documented in preterm piglets, a well-established model for preterm infants [9]. When compared with human donor milk, BC showed similar efficacy in decreasing incidence of NEC in pigs [10]. When used as minimal enteral nutrition (MEN) just after preterm birth, BC markedly improved intestinal digestive and immune functions, and prevented preterm pigs against NEC, relative to IF [11]. This indicates that BC may be used as MEN for preterm infants while waiting for mothers to lactate.

Currently, the first clinical pilot trial is ongoing to investigate the safety and tolerability of BC as the first enteral feeding for preterm infants and its primary effects on feeding tolerance and intestinal functions, relative to IF and donor human milk (ClinicalTrials.gov, NCT02054091). The first part of the study (twelve infants recruited) showed that BC was safe and well-tolerated [12]. If this study shows promising results, further research is warranted to explore whether it can be used to prevent NEC in PVLBW infants.

## 3. Probiotics

During the past decades, probiotics have been studied extensively in the prevention of NEC. Six recent meta-analyses confirmed the effectiveness of oral probiotics in reducing the incidences of NEC and death [13,14,15,16,17,18]. Many level III neonatal centers in Finland, Italy and Japan have been routinely using probiotics for over a decade and have not reported any significant adverse effects. However, clinicians are still concerned about the efficacy and potential adverse effects and are facing challenges in assessing which probiotics (or probiotic combinations) are the most effective ones for PVLBW infants. Published studies have used a variety of different single or combined probiotic strains with different target populations. Little is known about whether single-strain probiotics or probiotic combinations are more effective in the prevention of NEC and death in PVLBW infants.

Recent articles have shown an association between NEC and a lack of gut microbiota diversity [19,20]. One review article suggested that probiotic combinations were more beneficial than single-strain probiotics for gut and immune functions [21]. An updated meta-analysis including 21 trials (own data, manuscript submitted) confirmed the preventive effects of probiotics on NEC and death showed in previous systematic reviews. The updated meta-analysis focused on PVLWB or preterm infants of ≤34 weeks gestation (a high-risk group for NEC or death), who had undergone enteral administration of probiotics commenced within the first seven days of life and continued for at least 28 days. Supported by the results from a premature rat model [22], the meta-analysis showed that relative to single-strain probiotics, probiotic combinations resulted in a marked reduction in NEC incidence, with a pooled odds ratio (OR) of 0.37 (95% CI, 0.25–0.54; *p* < 0.00001) and mortality, with a pooled OR of 0.58 (95% CI, 0.43–0.79; *p* = 0.0006). The potential protective mechanisms might include increased diversity of the intestinal microbiota, and perhaps suggest the potential benefit of offering healthy bacteria such as Lactobacillus and Bifidobacterium to balance normal microbiota in this vulnerable population.

Based on the research evidence provided by randomized controlled trials (RCTs) and meta-analyses, probiotics should be offered routinely to preterm infants at high risk of NEC, if safe and clinically effective products are available.

## 4. Human Milk Oligosaccharides and Other Prebiotics

Human milk oligosaccharides (HMOs) are a family of structurally diverse glycans, which consists of more than one hundred substances and presents in human milk at concentrations up to 20 g/L [23]. Studies in animals suggest that HMOs play a role in many important biological functions, such as shaping intestinal microbiota composition as metabolic substrates [24,25], inhibiting the binding of pathogens to the mucosal epithelium as soluble decoy receptors [26,27], and dampening excessive mucosal leukocyte infiltration and activation as modulators [23,25,28,29]. However, studies showed that HMOs may favor clostridial population in the distal part of the intestine when fed to mice, and feeding sialyl(α2,3)lactose to interleukin 10-deficient mice increased colitis severity [30,31].

Recent rat studies showed that protective effects of HMOs against NEC were due to a specific isomer of disialyllacto-*N*-tetraose (DSLNT), and a synthetic HMO-mimicking prebiotics, galacto-oligosaccharides (GOS), had no effects in NEC prevention. This indicates that the protective effect of HMOs against NEC may be highly structure-specific, as GOS is very different from DSLNT in its chemical structure [29]. However, DSLNT is not easily obtained by either purification or synthesis due to the limited availability of human milk and low abundance in bovine milk [32]. Two novel disialyl hexasaccharides, disialyllacto-*N*-neotetraose (DSLNnT) and α2-6-linked disialyllacto-*N*-tetraose (DS’LNT), are readily available by enzymatic synthesis. They have been shown to protect neonatal rats against NEC [28]. One clinical study indicated that low concentrations of DSLNT in 4-day mother’s milk were associated with increased risk of NEC in PVLBW infants with HIV-infected mothers (200 ± 126 *vs.* 345 ± 186 μg/mL; *p* < 0.05) [33].

Effects of other prebiotics, such as GOS, fructo oligosaccharide (FOS), lactulose, and inulin, have been studied in clinical trials. The most recent meta-analysis on prebiotics showed no effects on NEC in preterm infants [24]. A multi-center study, ProPre-Save further confirmed that using prebiotics (inulin) alone failed to reduce the incidence of NEC (Bell stage ≥2) in PVLBW infants compared with placebo (12% *vs.* 18%; *p* > 0.05), although it had positive effects on feeding tolerance, sepsis, and mortality [34]. A recent study showed that addition of GOS/FOS mixture to breast milk significantly reduced the incidence of NEC (4.0% *vs.* 22.0%; hazard ratio: 0.49 (95% CI: 0.29–0.84); *p* = 0.002) and time to full enteral feeds (average of 11 (7–21) *vs.* average of 14 (8–36) days; *p* = 0.02) in exclusively breast-milk fed PVLBW infants [35]. However, in this study only one infant developed NEC with Bell stage >1, which indirectly supports the findings of the meta-analysis and the ProPre-Save study. More animal studies and clinical RCTs are needed to fully evaluate the effects of HMOs including DSLNnT and DS’LNT as promising therapeutic candidates in NEC prevention.

## 5. Long Chain Polyunsaturated Fatty Acids

Apart from the nutritional value for visual and cognitive development, *n-*3 long chain polyunsaturated fatty acids (LCPUFAs, e.g., docosahexaenoic acid, DHA) and *n-6* LCPUFAs (e.g., arachidonic acid, AA) have versatile biological effects on immune-modulation and inflammatory response [36]. Since nearly 90% of fetal fat deposition occurs during the last 10 weeks of gestation, and accretion of LCPUFAs increases markedly during the last trimester of gestation, earlier premature birth implies greater LCPUFA deficiency and greater need for LCPUFA supplementation.

LCPUFA supplementation reduced the incidence of NEC in an experimental neonatal rat model of NEC [37]. This effect may be due to the functions in maintaining the epithelial integrity, reducing bacterial and endotoxin translocation, and decreasing mucosal platelet-activating factor synthesis and receptor activation [38]. Furthermore, DHA has been shown to reduce lipopolysaccharide-induced nuclear factor (NF)-κB activation and IL-6 production in mice [39]. A recent study also showed that *n-*3 fatty acids are beneficial for protecting the premature intestine from inflammation by regulating eicosanoid- and NF-κB-related metabolite expression in premature rat pups [40]. These results suggest that LCPUFAs modulate various key factors involved in experimental NEC pathogenesis and partially explain why LCPUFAs have the protective effect on neonatal NEC.

In preterm infants, decreased postnatal DHA and AA in blood hads been associated with neonatal morbidities [41]. A recent meta-analysis disclosed that *n-*3 LCPUFAs supplementation were associated with a trend toward reduced risk of NEC (pooled relative risk 0.50, 95% CI 0.23–1.10, five studies, *n* = 900 infants) in infants born at ≤32 weeks gestation without detrimental effect [42]. Large-scale interventional studies are still required to define the clinical benefits of LCPUFA in PVLBW infants.

## 6. Glutamine

Glutamine (Gln) is an important nutrient for intestinal cell proliferation and small intestinal growth [43]. Studies in cell and animal experiments indicated that Gln exerts multiple biological activities such as antioxidant, anti-apoptosis, and anti-inflammation, which are involved in the pathophysiological mechanism of NEC [44,45]. Enteral supplementation of Gln attenuated local intestinal inflammatory damage in rats with NEC [43]. A recent study also found that Gln markedly reduced the mucosal injury by suppressing the expression of toll-like receptor (TLR) 2/4 and caspace-3 in the ileum and colon of neonatal rats with NEC, as TLRs play key roles in the pathogenesis of NEC [46,47].

Gln supplementation has been investigated in premature infants in a wide variety of clinical settings. Gln-supplemented parenteral nutrition (PN) for PVLBW infants has been showed to decrease NEC incidence compared with a standard PN solution (0/25 *vs.* 5/30; *p* < 0.01) [48]. However, meta-analysis and several subsequent trials did not verify the effect [49,50,51]. A recent study showed that enteral supplementation of Gln was safe and could significantly reduce feeding intolerance (*p* = 0.015) in PVLBW infants in the first days or weeks of life. There was also a tendency towards lowered risk of NEC and intestinal perforation, but the differences did not reach statistical significance [52].

The discrepancy between pronounced protective effects in animal models and negative results in clinical trials warrants further well-designed multicenter RCTs with adequately powered sample size to clarify the role of Gln as a preventive agent against NEC in PVLBW infants.

## 7. Cysteine and *N*-Acetylcysteine

*N*-acetylcysteine (NAC), the precursor of cysteine, is an important component in the production of intracellular glutathione [53]. Premedication with NAC was associated with less severe NEC lesions in an intraluminal casein-induced NEC model using neonatal piglets [54]. In another neonatal rat model of NEC, NAC showed a protective effect on intestinal injury through its anti-inflammatory and antioxidant properties [55]. This effect was also demonstrated in two recent studies in experimental animals [56,57].

Few trials have assessed the effects of cysteine or NAC on NEC in preterm infants as primary outcomes. One Cochrane review concluded that routine addition of short-term cysteine treatment improves nitrogen balance in preterm infants, but it does not support routine NAC supplementation for PVLBW infants. Thus, more investigations are needed to evaluate whether cysteine or NAC supplementation affects NEC outcomes in preterm infants [58].

## 8. l-Arginine and l-Citrulline

**Figure 2:**
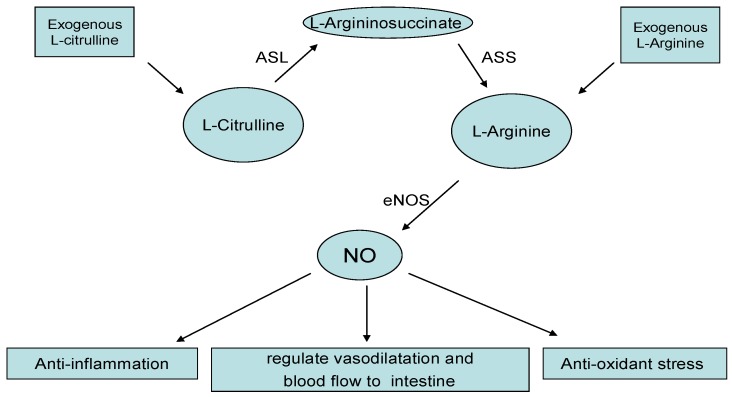
Possible mechanisms involved in the pathogenesis of Necrotizing enterocolitis (NEC). ASS, argininosuccinate synthase; ASL, argininosuccinate lyase; NO, nitric oxide; NOS, nitric oxide synthase.

Nitric oxide (NO) plays an essential role in NEC development by regulating vasodilatation and blood flow to the intestine [59,60]. Endogenous NO originates from the metabolism of l-arginine and l-citrulline, indicating their potential effects on NEC prevention (Figure 2). Low citrulline concentration has been reported in preterm infants [61,62] and piglets with NEC [63]. However, a recent study reported that low citrulline concentration in routinely collected neonatal dried blood spots was not associated with NEC [64]. There is no animal or clinical study assessing citrulline supplementation on NEC prevention.

Arginine, an essential amino acid for neonates, is exclusively synthesized by intestinal epithelial cells [65]. Low levels of plasma arginine in preterm infants are associated with increased incidence of NEC [62,66]. In several animal models of NEC, l-arginine supplementation has been proven to play pivotal roles in attenuation of intestinal injury by the l-arginine-NO pathway. For example, in the neonatal piglet NEC model, intravenous infusion of l-arginine markedly alleviated intestinal injury [67], and enteral supplementation of l-arginin increased intestinal mucosal growth [65]. In another ischemia/reperfusion induced NEC model in mice, dietary supplementation with l-arginine and l-carnitine attenuated the histological intestinal injury and significantly decreased lipid peroxidation in bowel injury [68].

Parenteral arginine supplementation in preterm infants increased plasma arginine levels and decreased NEC (6.7% *vs.* 27.3%; *p* < 0.01) in premature infants [69]. However, the outcome was confounded by the presence of many stage I NEC cases in the control group. In another double-blind RCT, enteral supplementation of l-arginine from day 3 to 28 after birth reduced the incidence of stage III NEC compared with placebo group [70]. A meta-analysis with 425 PVLBW infants enrolled showed a 60% reduction in all stages of NEC (RR 0.40, 95% CI 0.23 to 0.69, NNT = 5, *p* = 0.001) by arginine supplementation compared with placebo and a 59% reduction in the incidence of stage II and III NEC (RR 0.41, 95% CI 0.20 to 0.85, number needed to treat = 9, *p* = 0.02). It was concluded that l-arginine supplementation appeared to be protective against NEC without adverse effects in preterm infants [71].

Based on these findings, l-arginine supplementation deserves to be considered as a novel and potentially cost-effective method to prevent NEC. However, large multi-center RCTs are needed before this can become a common practice.

## 9. Discussion

NEC is a multifactorial disease with relatively high mortality, and its pathophysiology remains unclear. Several factors appear to contribute to the development of NEC, including immaturity of multiple intestinal functions, altered anti-inflammatory control, abnormal gut bacterial colonization, inappropriate enteral feeding and impaired host defense. The above-discussed immunonutrients have been shown in animal models to exert various physiological effects on intestinal growth and development, intestinal defense, immune-modulation and inflammatory response. These promising findings point toward the application of these nutrients as useful clinical tools. Some of these nutrients have been applied in clinical practices (e.g., probiotics) while others are still in the research stage (e.g., bovine colostrum). Although the effectiveness of probiotics in the prevention of NEC has been confirmed by various clinical trials and meta-analyses, there are still remaining questions in terms of the optimal strain or strain combinations, timing, dose, and duration of therapy. Further comparative studies are required to provide better clinical guidelines for probiotic therapy in preterm infants.

## 10. Conclusions

Enteral supplementation of probiotics is evident in NEC prevention, but clinical guidelines in terms of timing, strains, dose, length of therapy, and contraindications are urgently needed for PVLBW. Bovine colostrum seems to be a promising diet in replacement of IF to stimulate the development of the immature intestine while waiting for the mother’s own milk during the first days of life. LCPUFAs and HMOs might be directly supplemented to human milk or used to produce better preterm IF for those who have limited access to human milk. The aforementioned amino acids and LCPUFA might also be added to parenteral nutrition preparations. Clearly, more animal studies and clinical trials are required to further investigate the biological functions and to verify the safety and effectiveness of supplementation with immunonutrients in NEC prevention in PVLBW infants before recommendation of their routine use in the clinics (Table 2).

**Table 2:** Evidences of nutritional supplementation in the prevention of NEC.

Nutrients	Clinical Trials (Authors, Reference No.)	Outcomes		
		Primary	Secondary
Bovine colostrum	Yanqi Li *et al.*, [12]	safe and well tolerated	↑ enteral protein intake
Probiotics	Alfaleh K *et al.*, 2010. [15]	↓severe NEC and mortality	no reduction on sepsis and days on TPN
	Deshpande G *et al.*, 2010. [16]	↓mortality and 30% NEC	no reduction on sepsis
	Wang Q *et al.*, 2012. [17]	↓NEC and mortality	no reduction on sepsis
	Alfaleh K *et al.*, 2014. [18]	↓NEC and mortality	no reduction on sepsis
HMOs or Prebiotics	Van Niekerk E *et al.*, 2014. [33]	low concentrations of DSLNT were associated with NEC↑	NA
	Dilli D *et al.*, 2015. [34]	failed to reduce NEC	↓time to full enteral feeding, sepsis, mortality and stays in NICU
	Armanian AM *et al.*, 2014. [35]	↓NEC	↓time to full enteral feeds and duration of hospitalization
LCPUFAs	Zhang P *et al.*, 2014. [42]	↓NEC in infants born at ≤32 weeks gestation	↓BPD in infants born at ≤32 weeks gestation
Glutamine	Bober-Olesińska K *et al.*, 2005. [48]	↓NEC	no reduction on sepsis and stays in NICU
	Sevastiadou S *et al.*, 2011. [50]	↓NEC	↓sepsis
	Tubman TR *et al.*, 2008. [49]	no effect on mortality	no effect on NEC, infection, time to full enteral nutrition, or duration of hospitalization
	Mohamad Ikram I *et al.*, 2011. [51]	no reduction on NEC	no reduction on sepsis, duration of ventilation, and NICU stay
	Pawlik, D *et al.*, 2011. [52]	↓Feeding intolerance	lower but no significant differences in NEC, sepsis and intestinal perforation
Cysteine or NAC	Soghier LM *et al.*, 2006. [58]	↑Nitrogen retention No differences on growth	no reduction on death, NEC, BPD, ROP, IVH, PVL
l-Arginine, l-citrulline	Amin HJ *et al.*, 2002. [69]	↓NEC, ↑plasma arginine levels	no differences in nutrient intake, plasma ammonia and amino acid concentrations
	Polycarpou E *et al.*, 2013. [70]	↓NEC stage III	NA
	Mitchell K *et al.*, 2014. [71]	↓60% all stage NEC and 59% NEC stage II and III	no difference in any neurodevelopmental disability at 3 years

NEC: necrotizing enterocolitis; TPN: total parenteral nutrition; BPD: bronchopulmonary dysplasia; NICU: neonatal intensive Crtinopathy of prematurity care unit; ROP: retinopathy of prematurity; IVH:intraventricular hemorrhage; PVL: periventricular leukomalacia.

## Abbreviations

Necrotizing enterocolitis (NEC); preterm very low birth weight infants (PVLBW); gastrointestinal tract (GIT); bovine colostrum (BC); infant formula (IF); human milk oligosaccharides (HMOs); disialyllacto-*N*-tetraose (DSLNT); randomized controlled trials (RCTs); long chain polyunsaturated fatty acids (LCPUFAs); docosahexaenoic acid (DHA); arachidonic acid (AA); glutamine (Gln); *n*-acetylcysteine (NAC); nitric oxide (NO); odds ratio (OR); confidence interval (CI).
